# Blood cultures at central line insertion: a comparison with peripheral venipuncture

**DOI:** 10.1186/cc9647

**Published:** 2011-03-11

**Authors:** S Stohl, S Benenson, S Sviri, C Block, C Sprung, P Levin

**Affiliations:** 1 Children's Hospital of Philadelphia, PA, USA; 2 Hadassah Hebrew University Medical Center, Jerusalem, Israel

## Introduction

The objective was to compare contamination rates of blood cultures obtained at central line (CVC) insertion with cultures obtained at peripheral venipuncture or arterial line (AL) insertion. Contamination of blood cultures adds to cost, length of hospital stay, and unnecessary antibiotic administration. As most contaminants come from patients' skin, obtaining blood cultures after skin disinfection and under strict sterile precautions during CVC insertion might reduce contamination rates.

## Methods

A retrospective analysis of all blood cultures taken in the general and medical ICUs of a tertiary academic hospital over 8 years. Positive blood cultures were categorized as growing contaminants (Bacillus species, Corynebacterium species, Propionibacterium species, non-pneumococcal α-hemolytic Streptococci, and single-culture isolates of coagulase-negative Staphylococci), or true pathogens (all other results). Results of CVC insertion cultures were compared with peripheral venipuncture and AL insertion cultures.

## Results

A total of 17,384 blood cultures including 3,389 (19.5%) CVC, 1,844 (10.6%) AL and 12,151 (69.9%) peripheral cultures were analyzed. CVC insertion cultures were contaminated more frequently than AL or peripheral cultures (455/3,389 (13.4%) CVC, 103/1,844 (5.6%) AL, and 755/12,151 (6.2%) peripheral cultures, *P *< 0.001 CVC vs. peripheral and CVC vs. AL). However, true pathogens were found more frequently in CVC insertion cultures (445/3,389 (13.1%) CVC, 192/1,844 (10.4%) AL and 1,112/12,151 (9.2%) peripheral cultures, *P *< 0.001 CVC vs. peripheral and CVC vs. AL). The contamination and true positive rates for each source were almost identical in each ICU. Although there was a general decrease in culture contaminants over 8 years, the proportion of contaminants in CVCs remained approximately double that found in peripheral cultures at all time points.

## Conclusions

In complete contrast to the expected findings, and despite superior sterile precautions, cultures taken at CVC insertion had a higher contamination rate than either peripheral or AL blood cultures. These data were consistent in two completely independent ICUs and in cultures obtained over 8 years. The higher contamination rate may be related to the increased skin and soft tissue manipulations performed during CVC insertion. The higher true positive rate in CVC insertion cultures may indicate that these cultures retain utility.

